# P2Y_12_ Inhibitor Monotherapy after Percutaneous Coronary Intervention

**DOI:** 10.3390/jcdd9100340

**Published:** 2022-10-06

**Authors:** Xuan Zhou, Dominick J. Angiolillo, Luis Ortega-Paz

**Affiliations:** 1 Division of Cardiology, University of Florida College of Medicine, Jacksonville, FL 32209, USA; 2 Department of Internal Medicine, University of Alabama at Birmingham Montgomery, Montgomery, AL 36116, USA

**Keywords:** P2Y_12_ inhibitor, monotherapy, percutaneous coronary intervention, dual antiplatelet therapy, high bleeding risk, high on-treatment platelet reactivity, randomized controlled trial

## Abstract

In patients with acute and chronic coronary artery disease undergoing percutaneous coronary intervention (PCI), dual antiplatelet therapy (DAPT) has been the cornerstone of pharmacotherapy for the past two decades. Although its antithrombotic benefit is well established, DAPT is associated with an increased risk of bleeding, which is independently associated with poor prognosis. The improvement of the safety profiles of drug-eluting stents has been critical in investigating and implementing shorter DAPT regimens. The introduction into clinical practice of newer generation oral P2Y_12_ inhibitors such as prasugrel and ticagrelor, which provide more potent and predictable platelet inhibition, has questioned the paradigm of standard DAPT durations after coronary stenting. Over the last five years, several trials have assessed the safety and efficacy of P2Y_12_ inhibitor monotherapy after a short course of DAPT in patients treated with PCI. Moreover, ongoing studies are testing the role of P2Y_12_ inhibitor monotherapy immediately after PCI in selected patients. In this review, we provide up-to-date evidence on the efficacy and safety of P2Y_12_ inhibitor monotherapy after a short period of DAPT compared to DAPT in patients undergoing PCI as well as outcomes associated with P2Y_12_ inhibitor monotherapy compared to aspirin for long-term prevention.

## 1. Introduction

Percutaneous coronary intervention (PCI) with stent implantation has emerged as the predominant revascularization strategy in patients with obstructive coronary artery disease (CAD) [1,2,3]. After PCI, antiplatelet therapy plays a pivotal role in preventing stent-related complications such as stent thrombosis and secondary prevention for non-stent-related ischemic events such as myocardial infarction (MI) and stroke [4,5,6]. The combination of aspirin and an oral P2Y_12_ receptor inhibitor, known as dual antiplatelet therapy (DAPT), has become the guideline-recommended standard strategy after PCI based on data derived from more than 35 randomized clinical trials (RCTs) [1,2,7,8,9,10]. 

Clopidogrel is the most prescribed oral P2Y_12_ inhibitor [11]. In particular, clopidogrel is the only guideline recommended P2Y_12_ inhibitor after PCI in patients with chronic coronary syndromes (CCS) [1,2,7,8]. However, clopidogrel is a prodrug that requires hepatic cytochrome P450 2C19 (CYP2C19) metabolism to its active form, which leads to high variability in its pharmacodynamic (PD) effects [12,13]. Importantly, patients who persist with high platelet reactivity (HPR) while on clopidogrel are at increased risk of thrombotic events after PCI [14]. Indeed, patients with acute coronary syndromes (ACS) are at increased risk for HPR. Thus, the newer generation P2Y_12_ inhibitors prasugrel and ticagrelor characterized by potent and predictable antiplatelet effects are preferred over clopidogrel as the standard of care in patients with ACS [1,2,9,15]. 

Even though the efficacy of DAPT is well established, it is also associated with an unavoidable increased risk of bleeding, which is associated with poor outcomes, including increased mortality [16]. Several investigations have led to defining the phenotype of patients more prone to bleeding, setting the foundation for introducing the high bleeding risk (HBR) concept [17]. In 2019, the Academic Research Consortium (ARC) formally defined HBR patients as those who are at risk of ≥4% of having type 3 or 5 bleeding according to the bleeding academic research consortium (BARC) or ≥1% of intracranial hemorrhage (ICH), both at 1 year [18]. Moreover, the ARC-HBR proposed a diagnostic criterion based on clinical and laboratory characteristics that has been classified into major and minor criteria, the presence of 1 major or 2 minor criteria are needed to fulfil the HBR definition.

**Figure 1:**
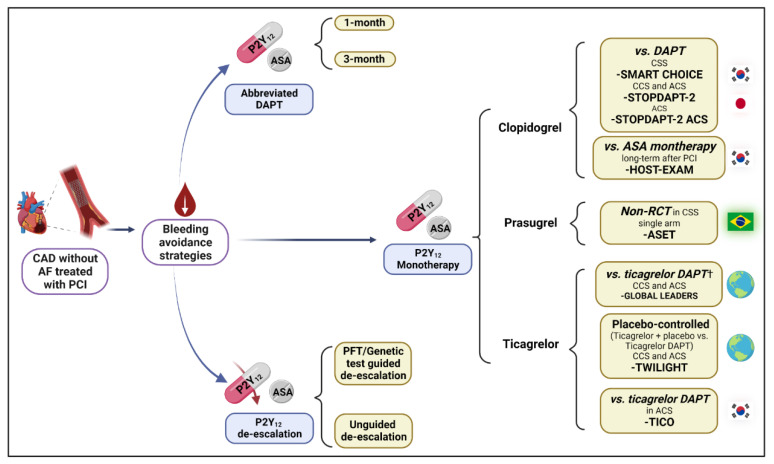
Selected bleeding avoidance strategies in patients without AF undergoing PCI. AF, atrial fibrillation; ACS, acute coronary syndrome; ASA, aspirin; CAD, coronary artery disease; CCS, chronic coronary syndrome; DAPT, dual antiplatelet therapy; PCI, percutaneous coronary intervention; PFT, platelet function test; RCT, randomized controlled trial.

Overall, these observations have prompted investigations evaluating “bleeding avoidance strategies” for patients undergoing PCI. The goal of these approaches is to minimize bleeding risk while preserving efficacy. Bleeding reduction strategies are directed to optimize the choice, duration, and modulation of DAPT (Figure 1). Amongst these, the strategy of discontinuation of aspirin after a short period of DAPT and maintaining P2Y_12_ inhibitor monotherapy has been a subject of extensive investigation. This strategy was first investigated in patients requiring concomitant use of an oral anticoagulant agent. The details of this approach go beyond the scope of this manuscript and are described elsewhere [19,20]. In this manuscript, we provide an overview of P2Y_12_ inhibitor monotherapy after a short course of DAPT in patients undergoing PCI without an indication of anticoagulation as well as the impact of P2Y_12_ inhibitor monotherapy compared to aspirin for long term secondary prevention in patients with CCS.

## 2. Rationale for P2Y_12_ Inhibitor Monotherapy

Platelet activation is a complex biological mechanism involving multiple activating factors such as thromboxane A_2_ and adenosine diphosphate (ADP), which represent the targets of aspirin and P2Y_12_ inhibitors, respectively [21]. Aspirin irreversibly blocks cyclooxygenase-1 (COX-1), the key enzyme in the arachidonic acid pathway of thromboxane A_2_ generation. On the other hand, P2Y_12_ inhibitors prevent ADP-mediated platelet activation by receptor blocking effect [22]. The exact mechanism can vary according to the type of drug. Clopidogrel and prasugrel (thienopyridines) require conversion to an active metabolite and mediate irreversible inhibition. Meanwhile, ticagrelor (nonthienopyridine) is a direct and reversible receptor antagonist [13]. Prasugrel and ticagrelor provide more potent and predictable platelet inhibition compared to clopidogrel [23,24]. These better PD profiles of prasugrel and ticagrelor compared to clopidogrel translate into lower ischemic/thrombotic events in pivotal RCTs, at the expense of increased bleeding events [25,26]. All these pivotal investigations have been performed on a background of aspirin therapy, under the notion that aspirin and P2Y_12_ inhibitors (mainly demonstrated with clopidogrel) have synergetic effects on platelet inhibition, representing the foundation for the use of DAPT [27,28]. 

Although DAPT has remained the standardized therapy after PCI, the usage and duration of aspirin have been challenged based on three major arguments. First, the synergism between aspirin and P2Y_12_ inhibitors was mainly established by early studies on aspirin with clopidogrel [28]. In the presence of potent P2Y_12_ blockade, in vitro pharmacodynamic investigations have shown that aspirin does not provide much additional antiplatelet effect [29]. This was also confirmed in a series of ex vivo pharmacodynamic studies [30,31,32]. While withdrawal of aspirin indeed eliminates its specific inhibitory effects mediated by the COX-1 pathway, other platelet signaling pathways are still affected by potent P2Y_12_ blockade [20,33]. Second, aspirin is associated with gastrointestinal (GI) adverse effects, from mild dyspepsia to ulceration and GI bleeding [34]. Systemically, aspirin irreversibly and non-selectively inhibits COX enzyme, leads to systemic prostaglandin depletion that compromises gastric mucosal barrier function and increases acid secretion [34]. Locally, aspirin may reduce surface hydrophobicity and destabilize the phospholipid barrier, which makes the mucosa susceptible to direct injury by gastric acid [35]. Although several approaches are used to mitigate aspirin gastric injury (i.e., consumption with food, proton pump inhibitors, and new aspirin formulations), the most effective way to reduce aspirin GI effects is by minimizing aspirin treatment duration [36]. Third, the introduction of newer drug-eluting stents has markedly decreased the rate of stent thrombosis, and the widespread usage of lipid-lowering therapies has further reduced the incidence of MI unrelated to the stent, which was assumed to be in part driven by the beneficial effects of DAPT [37]. 

## 3. Current Evidence of P2Y_12_ Inhibitor Monotherapy

**Figure 2:**
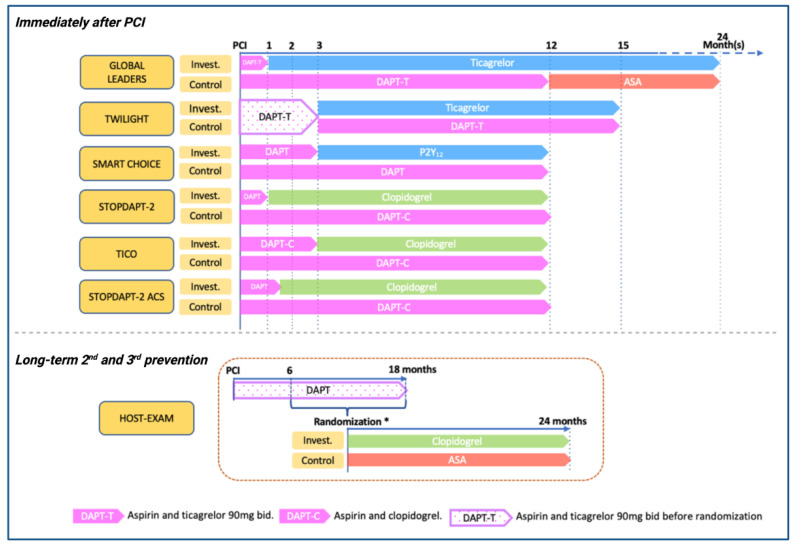
Randomized controlled trials of P2Y_12_ inhibitor monotherapy in patients treated with PCI. ASA, aspirin; DAPT, dual antiplatelet therapy; DAPT-C, clopidogrel-based dual antiplatelet therapy; DAPT-T, ticagrelor-based dual antiplatelet therapy; Invest., investigational group; PCI, percutaneous coronary intervention. * In HOST-EXAM trial, event-free patients who maintained DAPT for 6–18 months after PCI were randomized.

Over the last years, several large-scale RCTs have assessed the safety and efficacy of aspirin-free antiplatelet strategies after coronary stenting (Figure 2 and Table 1). Two main approaches have been assessed: (a) trials comparing P2Y_12_ monotherapy versus conventional DAPT regimens after PCI and (b) trials comparing P2Y_12_ inhibitors vs aspirin monotherapy for long-term secondary prevention. 

## 4. P2Y_12_ Monotherapy versus DAPT after PCI

### 4.1. Clopidogrel

SMART-CHOICE (Comparison Between P2Y_12_ Antagonist Monotherapy vs Dual Antiplatelet Therapy in Patients Undergoing Implantation of Coronary Drug-Eluting Stents) was an open-label RCT comparing 3-month DAPT followed by P2Y_12_ inhibitor monotherapy vs. standard 12-month DAPT after PCI in terms of major adverse cardiac and cerebrovascular events (MACCE) in a non-inferiority analysis [38]. A total of 2993 patients were enrolled. There were no restrictions on the type of P2Y_12_ inhibitor or clinical presentation. The P2Y_12_ inhibitor monotherapy was noninferior compared to DAPT in MACCE (Hazard ratio [HR], 1.19; 95% Confidence interval [CI], [−∞%–1.3%]; *p*_noninferiority_ = 0.007). There were no significant differences in the primary endpoint components, but there was a significantly lower BARC 2–5 bleeding rate in the P2Y_12_ inhibitor monotherapy than the DAPT group (HR, 0.58; 95%CI [0.36–0.92]; *p* = 0.020).

Two main post-hoc analyses have been reported. First, the clopidogrel–only cohort (80% of the total sample size), there were no significant differences between clopidogrel monotherapy versus clopidogrel–based DAPT in MACCE (HR, 1.02; 95%CI, [0.64–1.65]; *p* = 0.100) and BARC 2–5 bleeding (HR, 0.71; 95%CI, [0.42–1.21]; *p* = 0.150) [39]. Second, in the platelet reactivity sub-study (*n* = 833), 108 (13.0%) patients had HPR who had a significantly increased risk of MACCE compared to those without HPR (8.7% vs. 1.5%; HR, 3.03; 95%CI, [1.06–8.69]; *p* = 0.038) [40]. However, the treatment effect of clopidogrel monotherapy for the 12-month MACCE was not significantly different compared with DAPT in patients with HPR or without HPR (HR, 0.71; 95%CI, [0.18–2.73]; *p* = 0.628 and HR, 2.58; 95%CI, [0.68–9.77]; *p* = 0.161; *p*_interaction_ = 0.170). These results suggest that the main driver of adverse events was the HPR status rather than the allocated treatment, denoting the importance of optimizing platelet inhibition [41]. 

STOPDAPT-2 (Short and Optimal Duration of Dual Antiplatelet Therapy After Everolimus-Eluting Cobalt–Chromium Stent) was a prospective, open-labeled RCT comparing 1 month of DAPT (clopidogrel or prasugrel 3.75 mg od) followed by clopidogrel monotherapy versus 12 months DAPT with aspirin and clopidogrel in patients who underwent PCI [42]. A total of 3045 participants were recruited. The primary endpoint was a composite of ischemic (cardiovascular death, MI, stroke, or stent thrombosis) and bleeding endpoints (Thrombolysis in Myocardial Infarction [TIMI] major or minor bleeding) at 12 months. Clopidogrel monotherapy group met the prespecified criteria for noninferiority and superiority compared to the standard DAPT (HR, 0.64; 95%CI, [0.42–0.98]; *p* < 0.001 for noninferiority, *p* = 0.04 for superiority). There was no difference in the ischemic endpoints (HR, 0.79; 95%CI, [0.49–1.29]; *p* = 0.340), but there was a significant lower bleeding rate in the clopidogrel monotherapy than 12 months of DAPT (HR, 0.26; 95%CI, [0.11–0.64]; *p* = 0.004).

STOPDAPT-2 ACS (Short and Optimal Duration of Dual Antiplatelet Therapy-2 Study for the Patients With ACS) trial was a prospective, open-label RCT with the same design as the STOPDAPT-2, but including only patients with ACS, the ACS cohorts of both trials were combined (3008 newly enrolled and 1161 pooled form previous trial, in total 4169 patients were randomized) [43]. At the 1-year follow-up, 1–2 months DAPT (aspirin and clopidogrel) followed by clopidogrel monotherapy failed to meet the noninferior criteria compared to the 12-month DAPT (HR, 1.44; 95%CI, [0.80–1.62]; *p*_noninferiority_ = 0.06). The rate of major bleeding was significantly lower in the monotherapy group compared to the DAPT (HR, 0.46; 95%CI, [0.23–0.94]; *p* = 0.03). However, there was a significant increase in MI in the monotherapy group compared to the DAPT group (HR, 1.91; 95%CI, [1.06–3.44]; *p* = 0.03). The underlying reasons for which there was an increased risk of adverse events in the ACS cohort in patients treated with monotherapy compared to standard DAPT remains unclear but may be likely attributed to the presence of HPR among patients treated with clopidogrel only and no added antiplatelet effect given the withdrawal of aspirin.

STOPDAPT-2 Total Cohort the STOPDAPT investigators performed a prespecified pooled STOPDAPT-2 and STOPDAPT-2-ACS (*n* = 5997 in total), the rationale for this pooled analysis was that in both trials there had a lower-than-expected event rate that could affect the trials results [44]. The authors followed the same methodology and endpoints as in the main trials. One-month DAPT was noninferior but not superior to 12-month DAPT for the primary endpoint (HR, 0.94; 95%CI, [0.70–1.27]; *p*_noninferiority_ = 0.001 and *p*_superiority_ = 0.68). There was no significant risk-difference for the cardiovascular endpoint between groups (HR, 1.24; 95% CI, [0.88–1.75]; *p* = 0.23), but one-month DAPT was associated with a lower risk of the bleeding than 12-month DAPT (HR, 0.38 95%CI, [0.21–0.70]; *p* = 0.002). When the results were analyzed according to clinical presentation (ACS vs. CCS), one-month DAPT was associated with a lower risk for major bleeding than 12-month DAPT in ACS or CCS patients (HR, 0.46; 95%CI, [0.23–0.94]; *p* = 0.03. and HR, 0.26; 95%CI, [0.09–0.79]; *p* = 0.02; *p*_interaction_ = 0.40), but there was a numerical increase in cardiovascular events in ACS patients, but not in CCS patients (HR, 1.50; 95%CI, [0.99–2.27]; *p* = 0.053, and HR, 0.74; 95%CI, [0.38–1.45]; *p* = 0.39; *p*_interaction_ = 0.08).

### 4.2. Prasugrel

ASET (Acetyl Salicylic Elimination Trial) was a pilot, prospective, open-label, single-arm non-randomized study assessing the safety of prasugrel monotherapy in patients with CCS. All participants (*n* = 201) were on standard DAPT at the time of the index PCI, after successful PCI with platinum-chromium everolimus-eluting stent (Pt-EES), aspirin was discontinued and prasugrel was loaded and maintained for 3 months [45]. The primary ischemic endpoint was the composite of cardiac death, spontaneous target vessel MI, or definite stent thrombosis. The primary bleeding endpoint was major bleeding. There was only one event (cardiac death following intracranial bleeding). The compelling results of the ASET trial should be interpreted in the light of its small and very selected population and low lesion complexity.

### 4.3. Ticagrelor

GLOBAL LEADERS (A Clinical Study Comparing Two Forms of Antiplatelet Therapy After Stent Implantation) trial was a prospective, open-label RCT. Patients were randomized after successful PCI with a biolimus A9-eluting stent to either aspirin plus 90 mg ticagrelor twice daily for 1 month, followed by 23 months of ticagrelor monotherapy (90 mg, twice daily) or standard DAPT with clopidogrel (for patients with stable CAD) or ticagrelor (for patients with ACS) for 12 months, followed by aspirin monotherapy for another 12 months. A total of 15,968 patients were enrolled. The primary efficacy endpoint was all-cause death or non-fatal new Q-wave MI, and the primary safety endpoint was major bleeding, defined as BARC 3 or 5 bleeding. At 2 years, ticagrelor monotherapy was not superior to standard DAPT for reducing the primary efficacy (RR, 0.87; 95%CI, [0.75–1.01]; *p* = 0.073) or safety endpoints (RR, 0.97; 95%CI, [0.78–1.20]; *p* = 0.770). The adherence rate at two years was 77.6% in the experimental group and 93.1% in the control group, consistent with the premature ticagrelor discontinuation rate (25%) observed in other studies and mainly related to adverse events such as bleeding and dyspnea [46,47]. 

One of the main limitations of the GLOBAL LEADERS trial was the lack of independent event adjudication. Therefore, the prespecified GLASSY (GLOBAL LEADERS Adjudication Sub-Study) study was conducted following the same methodology as the main trial [48]. The study included approximately 47% of the main trial sample size enrolled in the top 20 enrolling sites. At 2 years, ticagrelor monotherapy was noninferior but not superior to standard 12 months DAPT for reducing the primary efficacy endpoint (RR, 0.85; 95%CI, [0.72–0.99]; *p*_noninferiority_ < 0.001 and *p*_superiority_ = 0.046 at alpha of 2.5%). There were no significant differences between groups in major bleeding regardless of the definition.

The prespecified [49,50,51,52,53,54,55,56] and selected post-hoc analyses [57,58,59,60,61] performed by the GLOBAL LEADERS investigators for exploring the effect size of the intervention on different subgroups are shown in Table S1.

TWILIGHT (Ticagrelor with Aspirin or Alone in High-Risk Patients after Coronary Intervention) was prospective, double-blind, placebo-controlled RCT that compared ticagrelor plus placebo vs. ticagrelor-based DAPT in event-free and high-risk PCI patients who completed 3 months of DAPT with aspirin and ticagrelor [62]. The primary endpoint was defined as clinically relevant bleeding (BARC 2, 3, or 5). The key secondary endpoint was the composite of all-cause death, nonfatal MI, or nonfatal stroke. A total of 7119 patient were randomized. At 1 year, the incidence of clinically relevant bleeding was significantly lower in the ticagrelor monotherapy group than in the ticagrelor-based DAPT group (HR, 0.56; 95%CI, [0.45–0.68]; *p* < 0.001). The secondary endpoint of BARC type 3 or 5 bleeding was also significantly less in the ticagrelor monotherapy group (HR, 0.49; 95%CI, [0.33–0.74]; *p* < 0.001). In the key secondary ischemic composite endpoint, ticagrelor monotherapy was non-inferior to ticagrelor-based DAPT group (HR, 0.99; 95%CI, [0.78–1.24]; *p*_noninferiority_ < 0.001). 

The main results of the TWILIGHT trial have been shown to be consistent in several subgroup analyses such as age [63], gender [64], East Asian ethnicity [65], DM status [66], CKD status [67], prior MI [68], clinical presentation [69], stent used [70], and HBR status [71]. Overall, all indicate a reduced risk of clinically relevant bleeding and without a significant increase in ischemic events. A complete list of the prespecified and post-hoc analyses performed by the TWILIGHT investigators are shown in Table S2.

TICO (Ticagrelor Monotherapy After 3 Months in the Patients Treated With New Generation Sirolimus-eluting Stent for Acute Coronary Syndrome) trial was prospective, open-label RCT comparing ticagrelor monotherapy after 3 months of DAPT versus ticagrelor-based DAPT for 12 months in patients with ACS treated with PCI [72]. The primary outcome was a net adverse clinical event (NACE, composite of MACCE [composite of all-cause death, MI, stent thrombosis, stroke, or target vessel revascularization] and TIMI major bleeding). A total of 3056 patients were randomized. At 1 year, ticagrelor monotherapy significantly reduced NACE compared to ticagrelor-based DAPT (HR, 0.66; 95%CI, [0.48–0.92]; *p* = 0.01). There was significant reduction in major bleeding between two groups (HR, 0.56; 95%CI, [0.34–0.91]; *p* = 0.02), but not in MACCE (HR, 0.69; 95%CI, [0.45–1.06]; *p* = 0.09).

The main results of the TICO trial have been shown to be consistent in several subgroup analyses such as DM status [73], high-ischemic risk [74], ST-segment elevation myocardial infarction (STEMI) [75], and HBR status [76]. A complete list of the prespecified and post-hoc analyses performed by the TICO investigators are shown in Table S3.

### 4.4. Meta-Analysis

Several meta-analyses have been reported. However, the most comprehensive data reported are the individual patient data metanalysis by Valgimigli et al. [77]. In total, 24,096 patients from the GLASSY, SMART-CHOICE, STOPDAPT-2, TICO, and TWILIGHT trials were included. The primary efficacy endpoint was defined as a composite of all-cause death, MI, and stroke, and the key safety endpoint was major bleeding (BARC type 3 or 5). In the intention-treat analysis, P2Y_12_ monotherapy was non-inferior but not superior to DAPT for the primary endpoint (HR, 0.93; 95%CI, [0.79–1.09]; *p* = 0.005 for noninferiority; *p* = 0.380). The bleeding risk was significantly lower with P2Y_12_ inhibitor monotherapy than DAPT (HR, 0.49; 95%CI, [0.39–0.63]; *p* < 0.001). In the subgroup analysis, there was a significant interaction of sex in the effect size of P2Y_12_ monotherapy and DAPT, there was a significant reduction in the primary endpoint in women but not in men (HR, 0.64; 95%CI, [0.46–0.89] and HR, 1.00; 95%CI, [0.83–1.19]; *p*_interaction_ = 0.02). The interaction was mainly driven by a reduction of cardiovascular mortality in women but not in men (HR, 0.31; 95%CI, [0.15–0.65] and HR, 0.86; 95%CI, [0.59–1.25]; *p*_interaction_ = 0.02). Furthermore, there was no significant interaction of the type of P2Y_12_ inhibitor (clopidogrel vs. newer P2Y_12_ inhibitor [mainly ticagrelor]) in the primary endpoint (HR, 0.94; 95%CI, [0.66–1.33] and HR, 0.89; 95%CI, [0.75–1.06]; *p*_interaction_ = 0.16) or major bleeding (HR, 0.60; 95%CI, [0.34–1.06] and HR, 0.47; 95%CI, [0.36–0.62]; *p*_interaction_ = 0.41).

## 5. P2Y_12_ Inhibitor versus Aspirin Monotherapy for Long-Term Secondary Prevention

CAPRIE (A Randomized Blinded Trial of Clopidogrel Versus Aspirin in Patients at Risk of Ischaemic Events) trial was a prospective double-blind RCT reported in 1996 comparing clopidogrel monotherapy with aspirin (325 mg daily) monotherapy in patients with atherosclerotic vascular disease (defined as recent ischemic stroke, recent MI, or symptomatic peripheral arterial disease) [78]. A total of 19,185 patients were enrolled with a mean follow-up of 1.91 years. The primary endpoint was a composite of ischemic stroke, MI, or vascular death, which was significantly lower in the clopidogrel monotherapy group than the aspirin group (relative risk reduction, 8.7%; 95%CI, [0.3–16.5]; *p* = 0.043). Clopidogrel monotherapy had a significant lower rate of gastrointestinal hemorrhage events (patients ever reporting: 2.0% vs. 2.7%; *p* < 0.05 and severe gastrointestinal hemorrhage: 0.5% vs. 0.7%; *p* < 0.05). Moreover, clopidogrel monotherapy had a better upper GI tolerability than aspirin alone, with significant less indigestion/nausea/vomiting reported (patients ever reporting: 15.0% vs. 17.56%; *p* < 0.05) [78]. Despite the benefits of clopidogrel over aspirin, aspirin has remained the mainstay of therapy considering its reduced costs with clopidogrel being recommended over aspirin only in patients who could not tolerate or with hypersensitivity to aspirin. However, over two decades later with the availability of generic formulations of clopidogrel, there has been a re-appraisal for P2Y_12_ inhibitor monotherapy for long-term secondary prevention. 

HOST-EXAM (Harmonizing Optimal Strategy for Treatment of Coronary Artery Stenosis-Extended Antiplatelet Monotherapy) trial was a prospective, open-label RCT comparing clopidogrel monotherapy or aspirin monotherapy for 24 months in event-free patients who were on DAPT for 6–18 months after PCI (*n* = 5530) [79]. The primary endpoint was a composite of all-cause death, non-fatal MI, stroke, readmission due to ACS, and major bleeding (BARC 3–5). At 2 years, clopidogrel monotherapy significantly reduced the primary endpoint compared to aspirin monotherapy (HR, 0.73; 95%CI, [0.59–0.90]; *p* = 0.003), driven by both the ischemic composite endpoint (HR, 0.68; 95%CI, [0.52–0.87]; *p* = 0.003) and major bleeding (HR, 0.63; 95%CI, [0.41–0.97]; *p* = 0.035).

GLOBAL LEADERS investigators performed a post-hoc landmark analysis between the first and second year of follow-up in patients who were event free during the first year [80]. In particular, during this period, patients were on ticagrelor monotherapy and aspirin monotherapy. There was a lower rate of MI in the ticagrelor monotherapy compared to the aspirin monotherapy group (adjusted HR, 0.74; 95%CI, [0.58–0.96]; *p* = 0.022), but at the expense of a higher rate of major bleeding (adjusted HR, 1.89; 95%CI, [1.03–3.45]; *p* = 0.005).

### Meta-Analysis

The P2Y_12_ inhibitor or aspirin monotherapy as secondary prevention in patients with coronary artery disease: an individual patient data meta-analysis of randomized trials (PANTHER) trial assessed the role of long-term P2Y_12_ monotherapy compared to aspirin monotherapy for the prevention of recurrent events in patients with CAD [81]. This analysis included 24,325 patients from seven RCTs. The primary endpoint was the composite of cardiovascular or vascular death, any non-fatal MI, and any non-fatal stroke. At a median of 557 days, P2Y_12_ monotherapy was associated with a significant reduction in the primary endpoint compared to aspirin monotherapy (HR, 0.88; 95%CI, [0.79–0.97]; *p* = 0.014). The P2Y_12_ monotherapy was associated with a significant reduction in MI (HR, 0.89; 95%CI, [0.81–0.98]; *p* = 0.020) and definite/probable stent thrombosis (HR, 0.46; 95%CI, [0.23–0.92]; *p* = 0.028) without a significant reduction in major bleedings (HR, 0.87; 95%CI, [0.70–1.09]; *p* = 0.230), and all cause-death (HR, 1.04; 95%CI, [0.91–1.20]; *p* = 0.560). Concerning the bleeding causes, P2Y_12_ monotherapy was associated with a significant reduction in gastrointestinal bleeding (HR, 0.75; 95%CI, [0.57–0.97]; *p* = 0.027) and ICH (HR, 0.32; 95%CI, [0.14–0.75]; *p* = 0.009).

## 6. Guidelines on P2Y_12_ Inhibitor Monotherapy

Several scientific societies have incorporated P2Y_12_ monotherapy among their recommendations in patients treated with PCI. The 2020 European Society of Cardiology (ESC) guidelines for the management of non-ST-elevation acute coronary syndrome (NSTE-ACS) recommend stopping aspirin after 3–6 months should be considered, depending on the balance between the ischemic and bleeding risk [9]. The 2021 American College of Cardiology (ACC), American Heart Association (AHA), and Society for Cardiovascular Angiography and Interventions (SCAI) guidelines for coronary artery revascularization which were developed after the ESC guidelines and thus had more data available, state that in selected patients undergoing PCI, shorter duration DAPT (1–3 months) is reasonable, with subsequent transition to P2Y_12_ inhibitor monotherapy to reduce the risk of bleeding events (Table 2) [2]. For long-term secondary prevention, clopidogrel is recommended in patients who cannot take aspirin due to intolerance or hypersensitivity [8]. 

## 7. Ongoing Studies of P2Y_12_ Inhibitor Monotherapy

**Figure 3:**
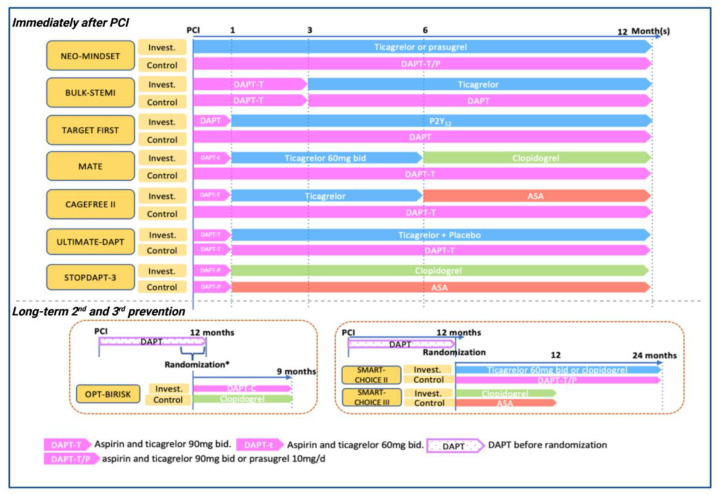
Ongoing randomized controlled trials of P2Y_12_ inhibitor monotherapy in patients treated with PCI. ASA, aspirin; DAPT, dual antiplatelet therapy; DAPT-C, clopidogrel-based antiplatelet therapy; DAPT-T, ticagrelor-based dual antiplatelet therapy; DAPT-T/P, ticagrelor-based or prasugrel-based dual antiplatelet therapy; Invest., investigational group; PCI, percutaneous coronary intervention. * OPT-BIRISK trial is randomizing patients with high ischemic or bleeding risk who already finished 9–12 months of DAPT.

The role of P2Y_12_ monotherapy in patients treated with PCI is currently a topic of extensive research with more than 10 ongoing RCTs (Table 3 and Figure 3). Overall, most of the ongoing trials are focused on ACS patients. In particular, ULTIMATE-DAPT is a placebo-controlled RCT that will recruit event-free patients after 1 month of DAPT and compare ticagrelor plus placebo or ticagrelor-based DAPT for 11 months. The MATE and CAGEFREE II trials are investigating a de-escalation strategy consisting of 1 month of DAPT, followed by 5 months of ticagrelor monotherapy, and finalized by 6 months of clopidogrel or aspirin monotherapy. Among HBR or ACS patients, STOPDAPT-3 will compare a short course if clopidogrel-based DAPT with standard clopidogrel DAPT duration. The BULK-STEMI will determine the efficacy of ticagrelor monotherapy after 3 months of ticagrelor-based DAPT in patients presenting with STEMI. Two studies, ASET-JAPAN and NEO-MINDSET, will also assess the role of prasugrel monotherapy, with peri-PCI aspirin only instead of short-term aspirin in other studies. Moreover, in the setting of prolonged antiplatelet therapy after a standard DAPT, SMART-CHOICE II, OPT-BIRISK, and SMART-CHOICE III trials will assess different long-term P2Y_12_ monotherapy regimens vs. DAPT or ASA monotherapy.

## 8. Gaps in Evidence

There are still several gaps in the knowledge that require further research. First, five out of seven trials studying P2Y_12_ monotherapy enrolled exclusively East Asian populations, who have lower ischemic risk and a higher tendency of serious bleeding than Caucasians (i.e., East Asian Paradox), limiting extrapolation of many of the study findings to other ethnicities [82]. Second, as a potent P2Y_12_ inhibitor, compared to ticagrelor, prasugrel has advantages including its once daily regimen and the less respiratory side effect, which greatly improves adherence. However, there are no dedicated RCTs of prasugrel monotherapy. Third, although HBR patients could benefit more from P2Y_12_ monotherapy as a bleeding reduction strategy, there are no dedicated RCTs in HBR patients and the current evidence is derived from post-hoc analysis. Fourth, four out seven trials used clopidogrel as the main P2Y_12_ inhibitor, platelet function testing or CYP2C19 genotyping to assess the probability of HPR was not performed in any of these trials and it is unclear if adverse events could be related to clopidogrel poor responders [41,83]. Ultimately, P2Y_12_ monotherapy has been mainly compared with standard DAPT regimens and it is unknown how this strategy compares with other bleeding avoidance strategies, including short DAPT with discontinuation of P2Y_12_ inhibitor and maintaining aspirin or de-escalation DAPT approaches (e.g., switching from ticagrelor/prasugrel to clopidogrel or reducing the dose of ticagrelor/prasugrel) [84]. The current gaps in knowledge and ongoing trials are summarized in Table 4.

## 9. Practical Implications

The P2Y_12_ monotherapy is an emerging strategy to be considered among the available bleeding avoidance strategies in selected patients taking into consideration the following. First, the safety and efficacy of monotherapy outside of RCTs are very limited, underscoring that the eligible patients are those who meet the specific selection criteria of the RCTs [85]. It should be underscored that these trials are heterogeneous in terms of enrolled populations (Western countries vs. East Asian countries) which could impact the thrombotic and bleeding risk profiles of the studied populations. Furthermore, previous studies have shown that different bleeding avoidance strategies (i.e., abbreviated DAPT vs. de-escalation) are associated with different impact on clinical outcomes, suggesting that the selected strategy should be tailored according to patient characteristics and desired outcomes [84]. Moreover, procedural characteristics could also raise the concern about the outcomes in patients treated with complex PCI. Nevertheless, post-hoc analyses of these trials have not shown impaired outcomes among patients treated with complex PCI [86]. Second, the clinical presentation and the selected P2Y_12_ inhibitor appear to impact outcomes. In particular, prasugrel, and ticagrelor are recommended over clopidogrel in patients with ACS. In the GLOBAL LEADERS, TWILIGHT, and TICO trials, patients with ACS treated with ticagrelor monotherapy reduced bleeding without affecting ischemic outcomes. However, in patients with ACS and clopidogrel monotherapy, the STOPDAPT-2 ACS trial showed reduced bleeding but increased ischemic events [43]. On the other hand, in CCS, clopidogrel appears to be a safe and effective drug, as shown in the SMART-CHOICE and STOPDAPT-2 trials [39,42]. Moreover, ticagrelor can also be an option in CCS with high ischemic risk as reported in the TWILIGHT trial [62]. Third, most of these trials were designed with run-in phases and randomized only event-free patients after a short course of DAPT (i.e., 1–3 months). Therefore, in daily clinical practice, the decision to drop aspirin and continue P2Y_12_ inhibitor monotherapy should be made according to these protocols. Ultimately, P2Y_12_ inhibitor monotherapy has been compared mainly with standard DAPT (i.e., guideline-recommended duration) up to one year after the index PCI or randomization. Therefore, the clinical benefit of P2Y_12_ inhibitor monotherapy compared to other DAPT regimens and beyond the following 12–15 months of PCI is uncertain. Nevertheless, the only recent piece of information about P2Y_12_ monotherapy for long-term 24 months in event-free patients who were on DAPT for 6–18 months after PCI) comes from the HOST-EXAM trial, which suggests that clopidogrel monotherapy is safe and effective strategy compared to aspirin monotherapy [79]. 

## 10. Conclusions

Although DAPT with aspirin and a P2Y_12_ inhibitor is the standard care and guideline-recommended strategy in patients treated with PCI, recent pharmacodynamic studies have shown limited synergistic effects of aspirin in addition to potent oral P2Y_12_ inhibitors and have challenged the need for DAPT to achieve optimal platelet inhibition. In fact, while DAPT is associated with a reduction in ischemic events, it also increases bleeding, the risk of which is proportional to the intensity and duration of DAPT. As thrombotic complications mostly occur early after PCI, while bleeding accrues over the time, bleeding reduction strategies have been developed so that enhanced antithrombotic effects are present in the early phases post-PCI end then reduced afterwards. To this extent, several RCTs have assessed the role of P2Y_12_ inhibitor monotherapy compared to a standard DAPT regimen. Overall, P2Y_12_ inhibitor monotherapy is safe and effective for reducing bleeding without compromising ischemic outcomes in event-free patients treated with PCI after a short course of DAPT. In particular, ticagrelor has shown optimal results in patients with ACS, whereas clopidogrel and ticagrelor have been safe and effective for preventing recurrent events in CCS. The P2Y_12_ inhibitor monotherapy has already been incorporated in European and American guidelines as a reasonable antiplatelet strategy in patients treated with PCI. Over ten RCTs are ongoing to confirm previous findings and provide new insights P2Y_12_ inhibitor monotherapy immediately after PCI, the role of prasugrel, and outcomes in patients with STEMI. Ultimately, ongoing research is warranted to define whether P2Y_12_ inhibitor monotherapy should be preferred over aspirin for long-term secondary prevention in patients with CCS.

## Figures and Tables

**Table 1:** Randomized controlled trials for P2Y_12_ inhibitor monotherapy in patients treated with PCI.

Studies	Experimental Group	Control Group *	Primary Outcome	Key Secondary Outcome
**Immediately after PCI**				
GLOBAL LEADERS 2018 (*n* = 15,968)	Ticagrelor-based DAPT for 1 month, then Ticagrelor monotherapy	ASA + clopidogrel (53%) ASA + ticagrelor (47%)	At 24 months, all-cause death, new Q-wave MI (RR, 0.87; 95% CI, [0.75–1.01]; *p* = 0.073)	BARC 3 or 5 bleeding (RR, 0.97; 95%CI, [0.78–1.20]; *p* = 0.770)
TWILIGHT 2019 (*n* = 7119)	Ticagrelor-based DAPT for 3 months, then Ticagrelor monotherapy	ASA + Ticagrelor	At 12 months, BARC 2–5 bleeding (HR, 0.56; 95% CI, [0.45–0.68]; *p* < 0.001)	BARC 3 or 5 bleeding (HR, 0.49; 95% CI, [0.33–0.74]; *p* < 0.001)
SMART CHOICE 2019 (*n* = 2993)	Clopidogrel (76.9%) Prasugrel (4.1%) Ticagrelor (19.0%) DAPT for 3 months, then monotherapy	ASA + clopidogrel (77.6%) ASA + Prasugrel (4.5%) ASA + ticagrelor (17.9%)	At 12 months, all-cause death, MI, stroke (difference, 0.4%; one-sided 95%CI, [−∞–1.3%]; *p* = 0.007 for non-inferiority)	BARC 2–5 bleeding (HR, 0.58; 95%CI [0.36–0.92]; *p* = 0.020)
STOPDAPT-2 2019 (*n* = 3045)	Clopidogrel based DAPT, then clopidogrel monotherapy	ASA + clopidogrel	At 12 months, CV death, MI, stroke, stent thrombosis, or TIMI major or minor bleeding (HR, 0.64; 95%CI, [0.42–0.98]; *p* < 0.001 for noninferiority; *p* = 0.04 for superiority)	TIMI major or minor bleeding (HR, 0.26; 95%CI, [0.11–0.64]; *p* = 0.004) -Ischemic endpoints (HR, 0.79; 95%CI, [0.49–1.29]; *p* = 0.340)
TICO (ACS) 2019 (*n* = 3056)	Ticagrelor-based DAPT, then ticagrelor monotherapy	ASA + ticagrelor	At 12 months, all-cause death, MI, stent thrombosis, stroke, target vessel revascularization and major bleeding (HR, 0.66; 95%CI, [0.48–0.92]; *p* = 0.01)	-TIMI major bleeding (HR, 0.56; 95%CI, [0.34–0.91]; *p* = 0.02) MACCE (HR, 0.69; 95%CI, [0.45–1.06]; *p* = 0.09)
STOPDAPT-2 ACS 2022 (*n* = 4169)	Clopidogrel-based DAPT, then Ticagrelor monotherapy	ASA + clopidogrel	At 12 months, CV death, MI, stroke, stent thrombosis, or TIMI major or minor bleeding (HR, 1.44; 95%CI, [0.80–1.62]; *p*_noninferiority_ = 0.06)	TIMI major or minor bleeding (HR, 0.46; 95%CI, [0.23–0.94]; *p* = 0.03) Significant increased risk of MI (HR, 1.91; 95%CI, [1.06–3.44]; *p* = 0.03)
**Long-term 2nd and 3rd prevention**				
HOST–EXAM 2020 (*n* = 5438)	Clopidogrel monotherapy, for 24 months	ASA monotherapy	At 24 months, all-cause death, non-fatal MI, stroke, readmission due to ACS, BARC 3–5 bleeding (HR, 0.73; 95%CI, [0.59–0.90]; *p* = 0.003)	BARC 3–5 bleeding (HR, 0.63; 95%CI, [0.41–0.97]; *p* = 0.035)

* Complete details about regimen duration are shown in Figure 1. ACS, acute coronary syndrome; ASA, aspirin; CAD, coronary artery disease; CCS, chronic coronary syndrome; CI, confidence interval; CV, cardiovascular; DAPT, dual antiplatelet therapy; HR, hazard ratio; PCI, percutaneous coronary intervention; PFT, platelet function test; RCT, randomized controlled trial; RR, rate ratio; TIMI, Thrombolysis in Myocardial Infarction.

**Table 2:** Clinical guidelines recommendations concerning P2Y_12_ inhibitor monotherapy.

Cardiology Societies	Clinical Scenario	Recommendations	Level of Evidence *	Class of Recommendation *
ESC	NSTE-ACS [10] (2020)	After stent implantation in patients undergoing a strategy of DAPT, stopping aspirin after 3–6 months should be considered, depending on the balance between the ischemic and bleeding risk.	IIa	A
	Chronic coronary syndrome [9] (2019)	Clopidogrel 75 mg daily is recommended as an alternative to aspirin in patients with aspirin intolerance.	I	B
ACC/AHA/SCAI	Coronary artery revascularization [2] (2021)	In selected patients undergoing PCI, shorter-duration DAPT (1–3 months) is reasonable, with subsequent transition to P2Y_12_ inhibitor monotherapy to reduce the risk of bleeding events.	A	2a

* Details of the specific methodology of level of evidence and class of recommendation are provided in each guideline. ESC, European Society of cardiology; American College of Cardiology, American Heart Association, and Society for Cardiovascular Angiography and Interventions; NSTE-ACS, non-ST elevation acute coronary syndrome; DAPT, dual antiplatelet therapy; PCI, percutaneous coronary intervention.

**Table 3:** Ongoing clinical trials for P2Y_12_ inhibitor monotherapy in patients undergoing PCI.

Studies	Design	Population	Experimental Group	Control Group	Primary Outcome	Key Secondary Outcomes
**RCTs immediately after PCI**						
NEO–MINDSET (*n* = 3400) (NCT04360720)	Open-label RCT 12 months follow-up	ACS	Ticagrelor or prasugrel monotherapy	ASA + ticagrelor or prasugrel	Ischemic: all-cause death, cerebrovascular accident, MI or urgent target vessel revascularization Bleeding: BARC type 2, 3 or 5	Stent thrombosis BARC 1–5 bleeding Cost-effectiveness ratio
ULTIMATE–DAPT (*n* = 3486) (NCT03971500)	Placebo-controlled RCT 12 months follow-up	No MACCE or major bleeding within 30 days	Ticagrelor and placebo for 11 months	ASA + ticagrelor for 11 months	MACCE, clinical-relevant bleeding (BARC ≥ 2), target vessel failure	Net adverse clinical events
STOPDAPT-3 (*n* = 3110) (NCT04609111)	Open-label RCT 12 months follow-up	Patients with HBR or ACS	ASA + prasugrel for 1 month followed by clopidogrel monotherapy 11 months	ASA + prasugrel 1 month, ASA monotherapy 11 months	BARC 3 or 5 bleeding; cardiovascular composite (cardiovascular death, MI, ischemic stroke, definite stent thrombosis)	Target lesion/vessel failure and revascularization
BULK–STEMI (*n* = 1002) (NCT04570345)	Open-label RCT 12 months follow-up	STEMI	Ticagrelor monotherapy after 3 months of DAPT (ASA + ticagrelor)	ASA + P2Y_12_ inhibitor after 3 months of DAPT (ASA + ticagrelor)	MACCE (all-cause death, MI, cerebrovascular event, stent thrombosis) and bleeding events (BARC 3 or 5)	
TARGET FIRST (*n* = 2246) (NCT04753749)	Open-label RCT 12 months follow-up	NSTEMI or STEMI with complete revascularization	P2Y_12_ monotherapy after 1 month of DAPT	12 months of DAPT	All-cause death, non-fatal MI, stent thrombosis, stroke, or bleeding events (BARC 3 or 5)	
MATE (*n* = 2856) (NCT04937699)	Open-label RCT 12 months follow-up	ACS and high bleeding risk	ASA + ticagrelor (60 mg bid) for 1 month → ticagrelor monotherapy (60 mg bid) for 5 months → clopidogrel monotherapy for 6 months	ASA+ ticagrelor	All-cause death, non-fatal MI, stroke, BARC type 2, 3 or 5 bleeding	
CAGEFREE II (*n* = 1908) (NCT04971356)	Open-label RCT 12 months follow-up	ACS treated with drug-coated balloon	ASA + ticagrelor for 1 month → ticagrelor monotherapy for 5 months → ASA monotherapy for 6 months	ASA + ticagrelor	All-cause death, stroke, MI, revascularization, BARC 3 or 5 bleeding	Stent thrombosis rates
**Non-randomized single-arm study**						
PIONEER IV CHINA (*n* = 285) (NCT05015699)	Open-label single arm 12 months follow-up	PCI with HT supreme DES	Ticagrelor monotherapy after 1 month of DAPT	None	All-cause death, stroke, MI, coronary revascularization	
ASET–JAPAN (*n* = 400) (NCT05117866)	Open-label single arm 3 months follow-up for CCS, 12 months for ACS	NSTE–ACS and CCS	Prasugrel (loading: 20 mg; maintenance: 3.75 mg/d) 3 months in CCS and 12 months in NSTE–ACS	None	Ischemic: cardiac death, target-vessel MI, definite stent thrombosis Bleeding: BARC 3 or 5 bleeding	
**Long-term 2nd and 3rd prevention**						
OPT–BIRISK (*n* = 7700) (NCT03431142)	Open-label RCT 9 months follow-up	ACS patients received 9–12 months of DAPT with high ischemic or bleeding risk	Clopidogrel for 9 months	ASA + clopidogrel for 9 months	BARC type 2–5 bleeding	MACCE
SMART–CHOICE II (*n* = 1520) (NCT03119012)	Open-label RCT 36 months follow-up after index procedure	No major MACCE at 12 month after BRS implantation	Clopidogrel or ticagrelor (60 mg bid) monotherapy for 24 months	ASA + clopidogrel or ticagrelor (60 mg bid)	Death, MI, cerebrovascular events	BARC 2, 3, 5 bleeding Revascularization Stent thrombosis
SMART–CHOICE III (*n* = 5000) (NCT04418479)	Open-label RCT 12 months follow-up	Patient finished 12 months of DAPT with high risk of recurrent ischemic events	Clopidogrel monotherapy	ASA monotherapy	MACCE	BARC 3/5 bleeding

ACS, acute coronary syndrome; ASA, aspirin; BARC, Bleed Academic Research Consortium; BRS, Bioresorbable scaffold; DAPT, dual antiplatelet therapy; HBR, high bleeding risk; MACCE, major adverse cardiac and cerebrovascular events; MI, myocardial infarction; RCT, randomized controlled trial; STEMI, ST elevation myocardial infarction; NSTEMI, non-ST-elevation myocardial infarction. The dosages without specific notes are: aspirin, 81–100 mg daily; ticagrelor, 90 mg twice daily; prasugrel, 10 mg daily.

**Table 4:** Current gaps in the evidence and potential research opportunities in the P2Y_12_ monotherapy.

Current Gaps	Ongoing Studies and Potential Research Opportunities
**Population:** Most recent clopidogrel monotherapy trials exclusively recruited Asian population, known to have different thrombotic and hemorrhaging profiles, thus limiting their external validity in western populations	
**Clinical presentation:** For ACS patients, data are controversial. In particular, the role of clopidogrel monotherapy. STEMI-focused trials are still needed	OPT–BIRISK, NEO–MINDSET, STOPDAPT-3, MATE, CAGEFREE II exclusively for ACS patients BULK–STEMI, TARGET FIRST use STEMI as a major inclusion criterion
**Specific conditions:** Studies on HBR patients are missing Dedicated trials assessing treatment for patients with on-treatment HPR are missing. Platelet function testing or CYP2C19 genotyping were not performed in clopidogrel trials	STOPDAPT-3 and MATE study HBR as inclusion criteria HPR-focused studies are warranted with deliciated platelet function test CYP2C19 genotyping needs to be performed in future clopidogrel trials
**Specific medications:** Data with prasugrel monotherapy is limited	NEO–MINDSET, ASET–JAPAN will include prasugrel monotherapy
**Comparison with other strategies:** It is unknown if P2Y_12_ monotherapy provides a significant benefit compared to other bleeding avoidance strategies (i.e., de-escalation or abbreviated DAPT regimens)	Dedicated RCTs are needed to compare clinical outcomes between patients treated with P2Y_12_ monotherapy vs. other bleeding avoidance strategies

ACS, acute coronary syndrome; DAPT, dual antiplatelet therapy; HBR, high bleeding risk; HPR, high platelet reactivity; RCT, randomized controlled trial; STEMI, ST elevation myocardial infarction; NSTEMI.

## Data Availability

Not applicable.

## Supplementary Materials

The following supporting information can be downloaded at: https://www.mdpi.com/article/10.3390/jcdd9100340/s1, Table S1: Prespecified and selected post-hoc analyses of GLOBAL LEADERS trial; Table S2: Prespecified and selected post-hoc analyses of TWILIGHT trial; Table S3: Prespecified and selected post-hoc analyses of TICO trial.

## Abbreviations

ACC	American College of Cardiology
ACS	acute coronary syndrome
ADP	adenosine diphosphate
AHA	American Heart Association
ARC	Academic Research Consortium
BARC	bleeding academic research consortium
BRS	bioresorbable scaffold
CAD	coronary artery disease
CCS	chronic coronary syndrome
CI	confidence interval
COX-1	cyclooxygenase-1
CV	cardiovascular
CYP2C19	hepatic cytochrome P450 2C19
DAPT	dual antiplatelet therapy
DAPT-C	clopidogrel-based dual antiplatelet therapy
DAPT-T	ticagrelor-based dual antiplatelet therapy
DAPT-T/P	ticagrelor-based or prasugrel-based dual antiplatelet therapy
DM	diabetes mellitus
ESC	European Society of Cardiology
GI	gastrointestinal
HBR	high bleeding risk
HPR	high platelet reactivity
HR	hazard ratio
MACCE	major adverse cardiac and cerebrovascular events
MI	myocardial infarction
NACE	net adverse clinical event
NSTE-ACS	non-ST-elevation acute coronary artery syndrome
PCI	percutaneous coronary intervention
PFT	platelet function test
POCE	patient-oriented composite endpoints
Pt-EES	platinum-chromium everolimus-eluting stent
RCT	randomized controlled trial
SCAI	Society for Cardiovascular Angiography and Interventions
SIHD	stable ischemic heart disease
STEMI	ST elevation myocardial infarction
TIMI	Thrombolysis in Myocardial Infarction
