# Supplementary material for: P2Y12 Inhibitor Monotherapy after Percutaneous Coronary Intervention
Source: J Cardiovasc Dev Dis. 2022 Oct 6;9(10):340. doi: 10.3390/jcdd9100340 (PMC9604207; doi:10.3390/jcdd9100340)
Supplement: Supplementary file 1 [file jcdd-09-00340-s001.zip › jcdd-1937575-supplementary.pdf]

Supplementary appendix

$P2Y_{12}$ Inhibitor Monotherapy after Percutaneous Coronary Intervention

Zhou et al.

Table of contents

Table S1. Prespecified and selected post-hoc analyses of GLOBAL LEADERS trial............2
Table S2. Prespecified and selected post-hoc analyses of TWILIGHT trial...........................3
Table S3. Prespecified and selected post-hoc analyses of TICO trial 4

Table S1. Prespecified and selected post-hoc analyses of GLOBAL LEADERS trial.

Sub study	Domain	Findings
Gao et al.[49]	Regional variation	Significant regional variance in the treatment effect of ticagrelor monotherapy was observed, which could be partially explained by the differences in PCI complexity.
Chichareon et al.[50]	Association of sex and outcomes	Women had a higher risk of bleeding and hemorrhagic stroke 2 years after PCI. Incidence of death and Q-wave MI did not differ between the sexes.
Tomaniak et al.[51]	Elderly patients (age ≥ 75)	There was no differential treatment effect of ticagrelor monotherapy found in elderly patients undergoing PCI concerning the rate of all-cause death or new Q-wave MI.
Ono et al.[52]	BMI with outcome (BMI < 27 vs. BMI ≥ 27)	BMI had no significant influence on the treatment effect of ticagrelor monotherapy. A beneficial effect of ticagrelor monotherapy was seen in ACS patients with BMI < 27 kg/m^2.
Chichareon et al.[53]	Diabetic patients	Diabetic patients had higher ischemic risk than non-diabetic patients, with similar bleeding risk. No significant differences in ischemic or bleeding outcomes were noticed between ticagrelor monotherapy and ticagrelor-based DAPT.
Tomaniak et al.[54]	Impaired renal function (IRF, eGFR < 60 mL/min/1.73 m^2)	At 2 years, patients with impaired renal function had a significantly higher rate of both ischemic and bleeding events than patients without IRF. There were no differential treatment effects between monotherapy and ticagrelor- DAPT in patients with IRF.
Kogame et al.[55]	Bifurcation PCI	There were no differences in patients with or without bifurcation PCI between ticagrelor monotherapy and ticagrelor-based DAPT. In patients with bifurcation PCI, ticagrelor monotherapy was associated with a significant reduction in stent thrombosis and increased stroke.
Gao et al.[56]	Radial vs. femoral access	At 30 days, there was no significant difference between radial and femoral access in ischemic events. Radial access had a significantly lower bleeding event. In high bleeding risk patients, radial access was associated with a lower ischemic and bleeding risk.
Franzone et al.[57]	SIHD vs. ACS patients	Ticagrelor monotherapy had consistent treatment effects on ischemic endpoint in patient with SIHD and ACS. Net clinical benefit was significant in ACS patients only.
Serruys et al.[58]	POCE and NACE analysis	Ticagrelor monotherapy was not associated with a significant reduction in POCE or NACE.
Takahashi et al.[59]	Long stenting (total stent length ≥ 46mm)	Ticagrelor monotherapy was associated with a significant reduction in ischemic risk without differences in bleeding risk and could potentially achieve an optimal NACE.
Chichareon et al.[60]	SYNTAX score	Overall discrimination for two-year all-cause mortality of the updated logistic clinical SYNTAX score is either borderline acceptable or helpful. Calibration in the majority of patients is appropriate.
Serruys et al.[61]	Complex PCI (defined as multivessel PCI, ≥3 stents, ≥3 lesions, bifurcation with ≥2stents, or total stent length >60mm)	In patients with complex PCI, ticagrelor monotherapy decreased ischemic risk and POCE; non-significant change of bleeding risk but significantly reduced NACE. Indicated a possible net clinical benefit.

GLOBAL LEADERS, A Clinical Study Comparing Two Forms of Antiplatelet Therapy After Stent Implantation; ACS, acute coronary syndrome; BMI, body mass index; GFR, glomerular filtration rate; IRF, imparted renal function; MI, myocardial infarction; NACE, net adverse clinical events; PCI, percutaneous coronary intervention; POCE, patient-oriented composite endpoints; SIHD, stable ischemic heart disease.

Table S2. Prespecified and selected post-hoc analyses of TWILIGHT trial.

Sub study	Domain	Findings
Angiolillo et al.[63]	Impact of age	Ticagrelor monotherapy significantly reduced bleeding risk among patients ≥ 65 years without increasing ischemic events. The beneficial effects are preserved irrespective of age.
Vogel et al.[64]	Sex differences and association with outcomes	The higher bleeding risk in women, mostly attributable to baseline differences, is no longer significant after multivariate adjustment. Ischemic events were similar between women and men.
Han et al.[65]	Influence of East Asian ethnicity	Ticagrelor monotherapy reduced bleeding without increasing ischemic events in Chinese patients.
Angiolillo et al. [66]	Impact of diabetes mellitus	There was a significant reduction in bleeding outcomes without an increase in ischemic events regardless of the diabetes mellitus status.
Stefanini et al.[67]	Impact of chronic kidney disease	Among CKD patients, ticagrelor monotherapy reduced the risk of bleeding without significantly increasing the ischemic risk.
Chiarito et al.[68]	Patients with prior MI	Ticagrelor monotherapy was associated with a significant lower risk of bleeding without increasing ischemic events in patients with prior MI.
Baber et al.[69]	Patients with ACS	Among patients with ACS, ticagrelor monotherapy had a more pronounced benefit of lower bleeding events without increasing ischemic risk.
Dangas et al.[70]	Impact of different DES on outcomes	Ticagrelor monotherapy was associated with reducing bleeding events without increasing ischemic risk, regardless of DES type.
Escaned et al.[71]	Outcomes in HBR patients	Among HBR patients, ticagrelor monotherapy significantly reduced bleeding events without increasing ischemic risk. The absolute risk reduction in major bleeding was larger in HBR than in non-HBR patients.

TWILIGHT, Ticagrelor with Aspirin or Alone in High-Risk Patients after Coronary Intervention; CKD, chronic kidney disease; DES, drug-eluting stent; DM, diabetes mellitus; HBR, high bleeding risk; MI, myocardial infarction; NSTE-ACS, non-ST-elevation acute coronary syndrome.

Table S3. Prespecified and selected post-hoc analyses of TICO trial

Sub study	Domain	Findings
Yun et al.[73]	Impact of diabetes mellitus	In diabetic patients, ticagrelor monotherapy had a lower incidence of bleeding without increasing ischemic complications.
Lee et al.[74]	High ischemic risk (defined as ≥ 3 stents; total stent length > 60mm; bifurcation with 2-stent; or history of DM or CKD)	There were no significant heterogeneities in the effect of ticagrelor monotherapy on outcomes in patients with or without high ischemic risk.
Lee et al.[75]	Patients with STEMI	Ticagrelor monotherapy was associated with lower incidence of bleeding without increasing ischemic complications in patients with STEMI.
Lee et al.[76]	Outcomes in HBR patients	Ticagrelor monotherapy associated with lower rate of adverse clinical outcomes regardless of HBR status, with similar magnitudes of therapy effect between HBR and non-HBR.

TICO, Ticagrelor Monotherapy After 3 Months in the Patients Treated With New Generation Sirolimus-eluting Stent for Acute Coronary Syndrome; CKD, chronic kidney disease; DM, diabetes mellitus; HBR, high bleeding risk; MACCE, major adverse cardiac and cerebrovascular events; STEMI, ST elevation myocardial infarction
